# Management of Pediatric Tibial Nonunion following Osteotomy: A Case Series and Review of the Literature

**DOI:** 10.1155/2020/8889066

**Published:** 2020-08-07

**Authors:** Gautham Prabhakar, Nicholas Kusnezov, Emmanuel Eisenstein, John C. Dunn, Amr Abdelgawad

**Affiliations:** ^1^Department of Orthopaedic Surgery, UT Health San Antonio, San Antonio, TX, USA; ^2^Department of Orthopaedic Surgery and Rehabilitation, Texas Tech University Health Sciences Center El Paso, El Paso, TX, USA; ^3^Department of Orthopaedics, Maimonides Medical Center, Brooklyn, NY, USA

## Abstract

Pediatric tibial nonunion following corrective osteotomy is a rare complication that is not well understood. While adult nonunions have been linked to endocrine and metabolic aberrations, this has not been established in a pediatric population. Pediatric tibial nonunion has been shown to respond to debridement with revision fixation using dynamic compression plating, supplementary bone graft, and fibular osteotomy to allow compression. Necessity of referral for metabolic and endocrinology workup remains unclear in the pediatric population, though inflammatory markers should be obtained in each case to rule out infection. We present three consecutive cases of pediatric tibial nonunion following osteotomy over a five-year period and discuss the management.

## 1. Introduction

Rotational and angular deformities of the lower extremity are common in the pediatric population. While most resolve spontaneously, occasionally, corrective osteotomy is required in the setting of persistence or progression of significant deformity, cosmetic abnormality, or functional disability [1–3].

There are a number of described surgical techniques for corrective tibial osteotomies utilized in the pediatric population that vary based on the plane of correction (derotational, angular, or combined), location (proximal metaphyseal, diaphyseal, or distal metaphyseal tibia), and type of fixation (percutaneous pins, plate and screws, and intramedullary device) [4–8]. The outcomes following these procedures are generally good and major complications are uncommon [1, 2, 6, 9–12]. Pediatric tibial nonunion following osteotomy is a rare complication that is not well understood [8, 10, 13–15]. While adult nonunions have been strongly linked to endocrine and metabolic aberrations, this has not been well established in a pediatric population [16]. There is likely a combination of biological and mechanical factors that contribute to pediatric nonunion. However, the thick periosteum and vital bone tissue seen in children provide greater osteogenic power than adults making this complication relatively rare. Nonetheless, the common belief that pediatric fractures heal readily may cause surgeons to treat this cohort less aggressively, which may yield unacceptable morbidity as seen in the current study [17].

There are presently no studies characterizing pediatric tibial nonunions following osteotomy. We present three consecutive cases of pediatric tibial nonunion following elective osteotomy for rotational and angular deformities of the lower extremity and discuss management.

## 2. Case Series

### 2.1. Case 1

The first case is that of a 3-year-old male with developmental delay and multiple musculoskeletal abnormalities including bilateral cleft feet who underwent right tibial supramalleolar derotational osteotomy for symptomatic external tibial torsion. The osteotomy was performed with an oscillating saw under irrigation and completed with an osteotome. A fibular osteotomy was performed at a different level. The tibia was derotated approximately 30 degrees, and fixation was obtained with three percutaneously placed 0.062 Kirschner wires.

**Figure 1:**
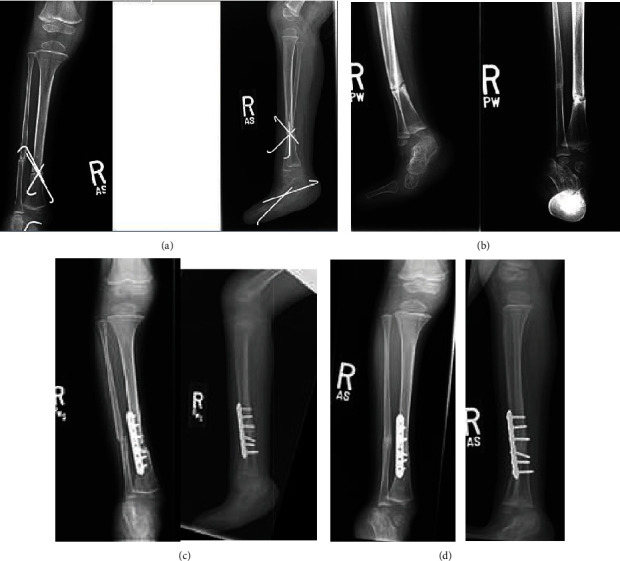
Supramalleolar derotational osteotomy in a 3-year-old male fixed with percutaneous 0.062-inch Kirschner wires. (a) Anteroposterior and lateral radiographs at 9-week postoperative time point demonstrating delayed healing of the tibial osteotomy site. (b) Six-month postoperative radiographs demonstrating no interval evidence healing at the tibial osteotomy site. (c) Immediate postoperative radiographs following revision open reduction with internal fixation of tibial nonunion. (d) Three-month postoperative radiographs showing union of the tibial osteotomy site.

Postoperatively, the patient was maintained in a cast with wires in place for 9 weeks, at which point, radiographs demonstrated complete consolidation of the fibular osteotomy with evidence of scant bridging callus at the tibial osteotomy site (Figure 1(a)). The bridging callus was thought to be an indication of healing; therefore, pins were removed and the patient was advanced to weight-bearing as tolerated. Unfortunately, subsequent follow-up failed to show progressive healing of the tibial osteotomy with concern for a delayed union. Laboratory workup was negative for infection or metabolic abnormality. The child was managed conservatively for a total of 6 months, and at the time of the 6-month follow-up, nonunion was diagnosed, as there had been no radiographic progression of healing during this period (Figure 1(b)).

The patient was taken back for revision open reduction with internal fixation, utilizing a 6-hole 2.7 mm plate in compression mode with supplemental demineralized bone matrix (Figure 1(c)). In order to achieve compression at the nonunion site, a concomitant fibular osteotomy was again created proximal to the tibial nonunion. Intraoperatively, the nonunion was found to be hypertrophic and had obliterated the medullary canal, which required drilling in order to reestablish one. Following revision surgery, the patient was immobilized in a cast for one month after which he was progressed to weight-bearing and went on to an uneventful union by the 3-month postoperative time point (Figure 1(d)). The patient later was taken back for hardware removal, Achilles tendon lengthening, and cleft foot reconstruction 28 months later. After healing of the nonunion, the relationship between the fibula and tibia was restored; however, the patient had abnormal anatomy including cleft foot with fused midfoot bones and calcaneus as well as an absence of the talus. At final follow-up two months status post his most recent surgery, the child was doing well with no complaints and a normal gait.

### 2.2. Case 2

**Figure 2:**
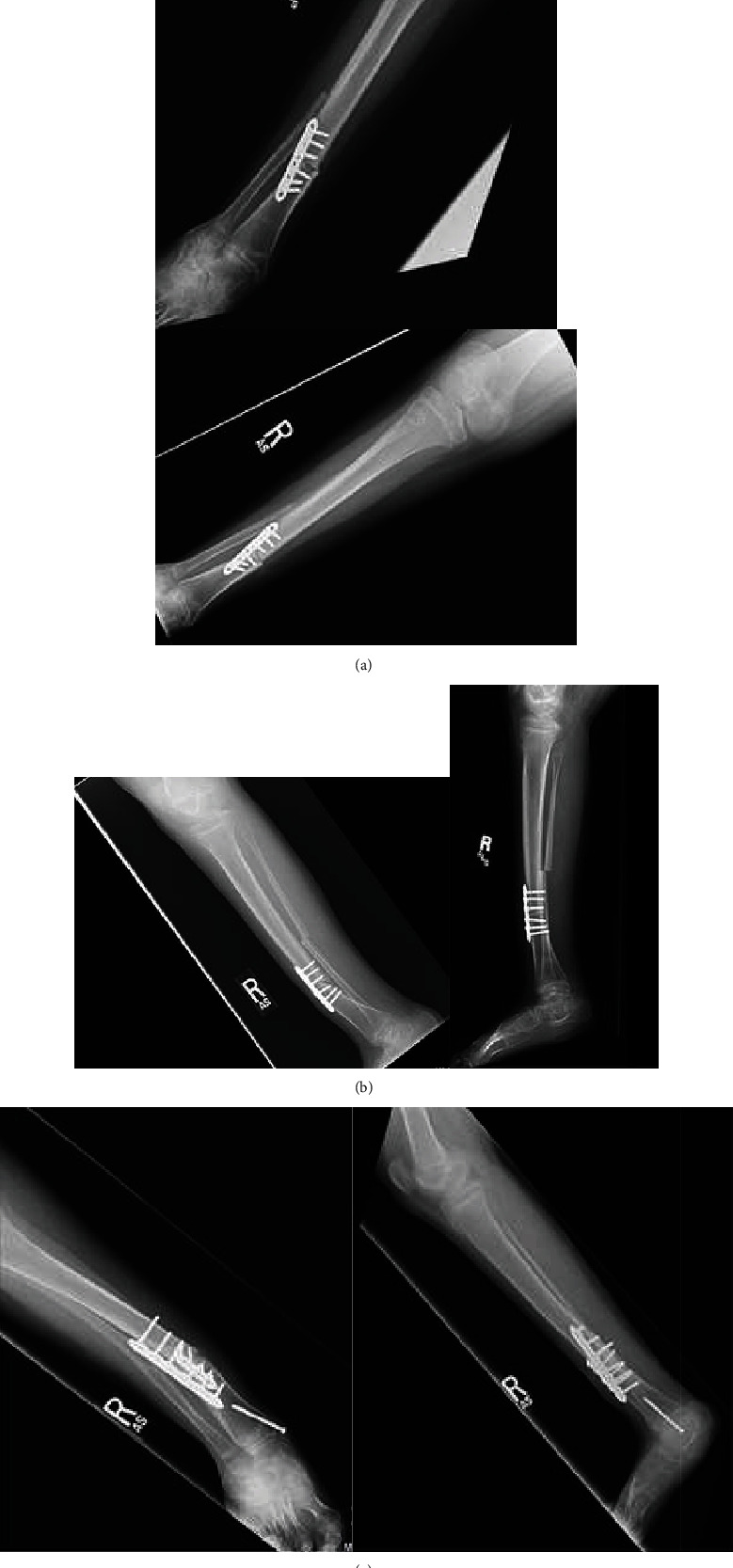
Supramalleolar derotational osteotomy in an 8-year-old male fixed with a 2.7 mm plate. (a) Postoperative anteroposterior and lateral radiographs. (b) Radiographs obtained at the 2-month postoperative visit demonstrating procurvatum and valgus angulation through the tibial osteotomy site. (c) Imaging obtained 14 weeks postrevision with perpendicular plating showing healing of osteotomy site with some residual valgus deformity.

The second case is that of an 8-year-old female with a history of juvenile rheumatoid arthritis, scleroderma, bilateral genu valgum, and right external tibial torsion who underwent supramalleolar 40-degree derotational osteotomy. The tibial osteotomy was performed with an oscillating saw under irrigation and completed with an osteotomy. Concomitant fibular osteotomy was again performed to permit adequate correction. Fixation was achieved with a 6-hole 2.7 mm plate in compression (Figure 2(a)). Concomitant bilateral distal medial femoral 8-plate hardware removal and extensor tendon releases to the lesser toes were performed.

Postoperatively, the patient was immobilized in a cast. At the 2-month postoperative visit, it was noted that the patient had been very active in her cast including jumping off the bed onto the ground resulting in procurvatum and valgus displacement at her osteotomy site (Figure 2(b)). The patient was treated with prolonged cast immobilization for the subsequent 4 months followed by continued immobilization in a walking boot for another 2 months, activity restriction, and the use of a bone stimulator. The patient was managed conservatively for a total of 10 months postoperatively, at which point, a computed tomography scan was obtained showing persistent hypertrophic nonunion at the tibial osteotomy site.

The patient was taken back for revision open reduction and internal fixation with correction of the apex anterior and valgus deformity. Intraoperatively, significant mobility was appreciated at the site of nonunion. Perpendicular plating was performed. A 4-hole 2.7 mm plate was placed anteriorly and a 6-hole 3.5 mm plate was placed laterally, both in compression mode. The patient was again cast-immobilized and made nonweight-bearing for the first 10 weeks, at which time she was transitioned to a walking boot and advanced to weight-bearing as tolerated. At this point, radiographs demonstrated bridging callus across three of four cortices at the osteotomy site. At her most recent follow-up 14 weeks status postrevision fixation, the patient had completely healed her osteotomy site, was asymptomatic, and had no activity restrictions (Figure 2(c)). We applied guided growth to control the remaining valgus deformity. As the patient had scleroderma with poor skin quality, minimal intervention was chosen in an attempt to avoid skin complications.

### 2.3. Case 3

The third and final case is that of a 16-year-old female with achondroplasia who underwent a varus-producing diaphyseal osteotomy for valgus deformity of the tibia. The osteotomy was performed with sharp osteotomes, and a concomitant fibular osteotomy was performed at a separate level to permit lengthening. Fixation was achieved via a Taylor spatial frame, and the deformity was corrected gradually.

**Figure 3:**
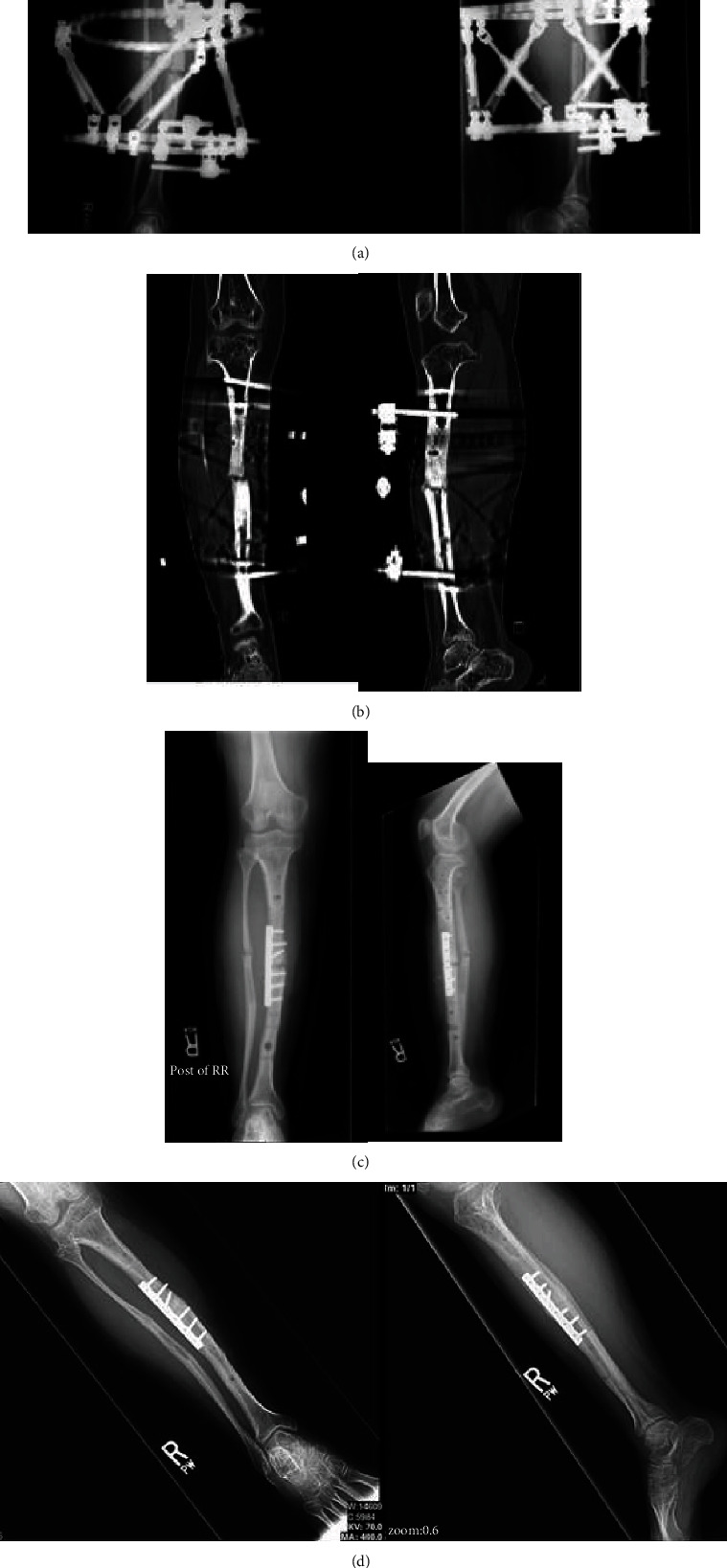
18-year-old female with achondroplasia who underwent a varus-producing diaphyseal osteotomy for valgus deformity of the tibia. (a) Anteroposterior and lateral radiographs demonstrating nonunion of diaphyseal osteotomy following Taylor spatial frame application for gradual corrective program. (b) Coronal and sagittal computed tomography reconstructions confirming nonunion of diaphyseal osteotomy despite completion of the program. (c) Revision open reduction and internal fixation with 3.5 mm plate 6 months following index frame application. (d) Radiographs obtained at 3 months following revision demonstrating union of the tibial osteotomy with no residual deformity.

Following completion of the program, the patient was allotted time for consolidation of the osteotomy site. However, at 6 months, the patient continued to experience persistent pain, which was worsened with ambulation. Radiographs demonstrated nonunion of the diaphyseal osteotomy (Figure 3(a)). This was confirmed by computed tomography (Figure 3(b)).

The patient was taken back at 6 months following the index procedure for the Taylor spatial frame removal and revision open reduction with internal fixation. Following debridement of the nonunion site, fixation was achieved with a 6-hole 3.5 mm plate in compression mode with supplementary demineralized bone matrix (Figure 3(c)).

The patient was placed in a walking cast and made weight-bearing as tolerated. The patient's pain gradually improved to the point of resolution, and the cast was removed at 6 weeks. She returned to regular activities by 3 months, at which time radiographs demonstrated complete consolidation of the osteotomy site (Figure 3(d)). At final follow-up 2 years following revision fixation, the patient remained asymptomatic.

## 3. Discussion

We report three consecutive cases of tibial nonunion following corrective osteotomy over a five-year period. The incidence of pediatric tibial nonunion following corrective osteotomy is undefined. Reported cases are exceedingly rare and appear to be largely independent of location, surgical technique, and type of fixation. A retrospective review of 55 closing-wedge varus-producing SMOs for progressive hindfoot valgus in patients with myelomeningocele reported one case of nonunion and two delayed unions in two patients who underwent bilateral SMOs [13]. Another large retrospective series of 247 percutaneous derotational SMOs with cast immobilization and early weight-bearing reported four delayed unions and only one nonunion [14]. These patients were older than 14 and with higher more metadiaphyseal osteotomies. Union was obtained in the one nonunion 8 weeks following reamed intramedullary nail fixation. Selber et al. presented an investigation of 91 derotational SMOs fixed with T-plates in patients with neuromuscular disease and reported only one aseptic nonunion attributable to a broken plate [10]. The patient achieved union following revision fixation with bone grafting. In the largest retrospective review, Payman et al. performed 129 tibial osteotomies in children with documented comorbidities including skeletal dysplasia and neuromuscular disease and reported only one infected nonunion following percutaneous pin fixation of a derotational SMO in an obese female [15]. The patient underwent reoperation with iliac crest bone graft and went on to uneventful union.

Given the rarity of pediatric tibial nonunion, risk factors and preventative measures are not well understood. Authors have suggested increasing age and diaphyseal osteotomy contribute to decreased potential for healing [14]. A series of 59 tibias treated with middiaphyseal osteotomy fixed with an intramedullary rod for isolated symptomatic tibial torsion reported one nonunion [8]. Though radiographs demonstrated apparent healing at 11 months, the patient went on to develop painless progressive bowing of the tibia over the subsequent 4 months and went again for revision intramedullary fixation. The increased cancellous surface area and abundant blood supply supports a more favorable environment following metaphyseal osteotomy. Interestingly, the majority of reported nonunions, including the two of our three, occurred following metaphyseal osteotomies. This may be confounded by the fact that middiaphyseal tibial osteotomies have largely been replaced by metaphyseal osteotomies today in most centers.

The point at which reoperation should be considered is furthermore undefined. Absolute indications would be implant failure with signs of incomplete healing and persistent pain or progressive deformity at the osteotomy site combined with radiographic evidence suggestive of nonunion. Given that radiographic evidence of union may be delayed for several months, especially in the setting of diaphyseal osteotomies, the authors recommend obtaining a computed tomography scan of the osteotomy site prior to pursuing revision fixation. Relative indications may include persistent pain with intact fixation and radiographic evidence of delayed union.

In adults, metabolic or endocrine abnormality has been identified in as many as 84% of patients with nonunion [16]. In this investigation, union was obtained in 30 of 31 patients following correction of the abnormality and subsequent revision fixation. Some authors recommended an endocrinology referral for all patients with (1) an unexplained nonunion occurring despite adequate reduction and stabilization, (2) history of multiple low-energy fractures with at least one progressing to nonunion, or (3) a nonunion or a nondisplaced pubic rami or sacral ala fracture [16]. In our three cases as well as all reported cases of nonunion following tibial osteotomy in the literature, patients responded to revision fixation with addition of autograft/allograft and went on to uneventful union [10, 14]. While we ruled out infected nonunion prior to revision surgery, we did not refer any of our patients for metabolic and endocrine workup. There was additionally no discussion of similar referral in any of the series reporting nonunion following tibial osteotomy [8, 10]. Therefore, it may be that underlying metabolic or endocrine abnormality is not as significant as a contributing factor to nonunion in children and may not be necessary. However, inflammatory markers should be obtained to rule out infected nonunion.

The cause for index nonunion remains unclear, though it may be attributable to poor local biology as all reported cases were atrophic or oligotrophic in nature. Revision construct varies widely among reported cases. In our cases, we debrided the nonunion site and utilized small frag plates in compression mode with supplemental bone graft. Therefore, the authors recommend the aforementioned construct with supplementation with bone graft at the time of revision fixation to achieve both a favorable biomechanical environment and biologic milieu.

## 4. Conclusion

Pediatric tibial nonunion after osteotomy is a rare complication yet can occur even in very young patients. Despite the excellent healing potential in children, the authors caution against less aggressive treatment options, as unacceptable morbidity such as nonunion may indeed occur. Continued pain and progressive deformity may suggest nonunion. Radiographs may furthermore be difficult to interpret initially given the hypertrophic nature of the nonunion and delayed evidence of radiographic healing; therefore, computed tomography may be required. Pediatric tibial nonunion has been shown to respond to debridement with revision fixation using dynamic compression plating, supplementary bone graft, and fibular osteotomy to allow compression. Necessity of referral for metabolic and endocrinology workup remains unclear in the pediatric population, though inflammatory markers should be obtained in each case to rule out infection.
